# Supplementary material for: MiT/TFE factors control ER‐phagy via transcriptional regulation of FAM134B
Source: EMBO J. 2020 Jul 27;39(17):e105696. doi: 10.15252/embj.2020105696 (PMC7459426; doi:10.15252/embj.2020105696)
Supplement: Supplementary file 1 — Expanded View Figures PDF [file EMBJ-39-e105696-s001.pdf]

# Expanded View Figures

**Figure EV1. FGF signaling modulates lysosome biogenesis and function.**

A FACS analysis of LysoTracker dye fluorescence in RCS chondrocytes treated with FGF ligands for 4 h (50 ng/ml). Fluorescence intensities were expressed as % relative to vehicle (5% ABS). Mean ± standard error of the mean (SEM) of $N$ = 3 biological replicates/treatment.

B FACS analysis of DQ-BSA dye fluorescence in RCS chondrocytes treated with the indicated FGF ligands (50 ng/ml; 4 h). Fluorescence intensities were expressed as % relative to vehicle (5% ABS). Concanamycin A (100 nM; 1 h) was used to inhibit lysosomal degradation. Mean ± standard error of the mean (SEM) of $N$ = 3 biological replicates.

C Immunofluorescence staining of Lamp1 (red) in RCS chondrocytes treated with vehicle (5% ABS) and FGF18 (50 ng/ml) for 4 h. Nuclei were stained with DAPI (blue). Scale bar 10 μm. Representative images of three independent experiments.

D Enzymatic assay of lysosomal β-glucuronidase and β-hexosaminidase enzymes in chondrocytes with indicated genotypes (ctrl = wild type) treated with FGF18 (50 ng/ml) for 16 h. Mean ± standard error of the mean (SEM) $N$ = 5 biological replicates (Gusb) and $N$ = 6 biological replicates (Hexb). One-way analysis of variance (ANOVA) $P < 0.001$; Tukey's post hoc test ***$P < 0.0005$; **$P < 0.05$; *$P < 0.05$; NS, not significant. Blank represents the value of the substrate alone.

E Western blot analysis of wild type (ctrl) and $ATG9A^{KO}$ RCS, showing the absence of ATG9A protein and SQSTM1/p62 accumulation in the KO compared to ctrl. Representative images of $N$ = 3 biological replicates. β-actin was used as a loading control.

F Co-immunofluorescence staining of ER (ER-Tracker BODIPY Green) and lysosomes (Lamp1, red) in $ATG9A^{KO}$ chondrocytes treated with 5% ABS (vehicle) or FGF18 (50 ng/ml) for 16 h. Representative images of $N$ = 3 biological replicates. Scale bar 10 μm.

G Western blot analysis of $FGFR^{KO}$ clones (ctrl = wild type) showing the absence of indicated FGFR proteins. β-actin was used as a loading control. Asterisks indicate non-specific bands.

H FACS analysis of LysoTracker dye fluorescence in RCS with indicated genotypes (ctrl = wild type) treated with FGF18 (50 ng/ml) for 16 h. Fluorescence intensities were expressed as % relative to vehicle (5% ABS). Mean ± standard error of the mean (SEM) of $N$ = 3 ($FGFR3^{KO}$, $FGFR4^{KO}$, $FGFR1^{KO}$, $FGFR3;1^{KO}$, $FGFR3;4^{KO}$) $N$ = 8 ($FGFR2^{KO}$) $N$ = 6 ($FGFR1;2^{KO}$) biological replicates/treatment/genotype. Analysis of variance (ANOVA) $P = 4.51e^{-5}$: Tukey's post hoc test ***$P < 0.0005$; **$P < 0.005$; *$P < 0.05$; NS, not significant.

I Representative immunofluorescence staining of Lamp1 (red) in chondrocytes with indicated genotypes. Higher magnification insets showed enlarged lysosomes in $FGFR3/4^{KO}$ chondrocytes. Nuclei were stained with DAPI (blue). Scale bar 15 and 5 μm (higher magnification boxes). Representative images of $N$ = 3 biological replicates/treatment.

J Representative TEM images of wild type (ctrl) and $FGFR3;4^{KO}$ chondrocytes showing lysosomes (arrows); scale bar 500 nm. Insets show enlargement of lysosomes; scale bar 250 nm. Quantification of organelle diameter (nm). Mean ± standard error of the mean (SEM) of $N$ = 3 biological replicates/genotype. Student's unpaired $t$-test ***$P < 0.0005$; NS, not significant. $n$ = 45 (ctrl) and $n$ = 51 ($FGFR3;4^{KO}$) cells were analyzed. Lys = lysosome; EE = endosome.

Source data are available online for this figure.

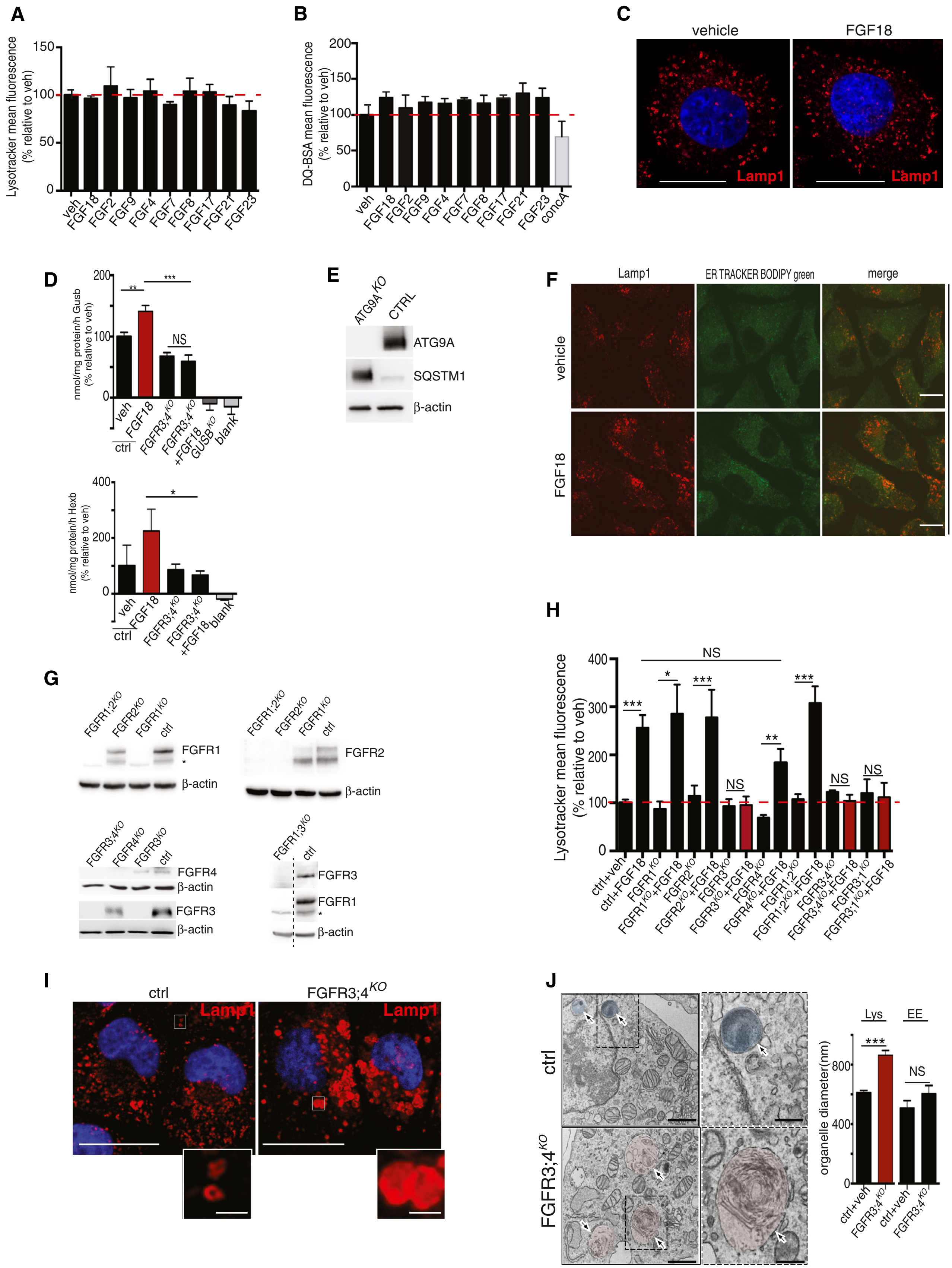
A
B
C
vehicle
FGF18
Lamp1
D
E
ATG9A
SQSTM1
β-actin
F
Lamp1
ER TRACKER BODIPY green
merge
vehicle
FGF18
H
Lysotracker mean fluorescence (% relative to veh)
G
FGFR1
FGFR2
FGFR4
FGFR3
I
ctrl
J
ctrl
organelle diameter (nm)
Lys
EE

Figure EV1.

**Figure EV2. FGF-dependent regulation of IRS1-PI3K signaling and of TFEB and TFE3 activity.**

A Western blot analysis of p-P70S6K (T389), P70S6K, p-4EBP1(S65), 4EBP1, p-S6 ribosomal protein (S240/S242), S6 ribosomal protein, p-AKT (S473), AKT proteins in RCS chondrocytes treated as indicated (FGF18: 50 ng/ml 16 h; Torin 1: 1 μM for 2 h; amino acid starvation (AA-) for 50 min; amino acid re-feeding (AA) : 3× for 20 min). Representative images of $N = 3$ biological replicates. β-actin was used as a loading control.

B Western blot analysis of IRS1 and phospho-IRS1 (S307) in chondrocytes treated with vehicle (5%ABS), FGF18 (50 ng/ml), and JNK inhibitor (50 μM) for indicated time. Filamin A was used as a loading control. Blot is representative of $N = 3$ independent experiments.

C KEGG pathway analysis of Quantsec gene expression analysis in RCS chondrocytes treated with vehicle (5% ABS) and FGF18 (50 ng/ml) overnight, showing upregulated (red) and downregulated (green) biological processes and cellular components.

D Schematic diagram showing qPCR primers used to analyze Fam134b isoform expressions. Arrows indicate the positions qPCR primer pairs used to detect Fam134b-1 (black arrows), Fam134b-2 (brown arrows), or both (turquoise arrows) Fam134b isoforms. Primer sequences are listed in *Materials and Methods*.

E TFEB (green) subcellular localization analysis in wild type (control) and FGFR3;4$^{KO}$ chondrocytes treated with FGF18 (50 ng/ml) for 16 h. Torin 1 was used at 1 μM for 2 h as positive control of TFEB nuclear translocation. Nuclei were stained with DAPI (blue). Scale bar 15 μm. Quantification is shown in Fig 3A.

F Chromatin immunoprecipitation experiment in TFEB-WT overexpressing cells treated with FGF18 (50 ng/ml) for 16 h, showing enrichment of TFEB binding on Mucolipin-1 promoter upon FGF18 treatment. $N = 3$ biological replicates. Mean ± standard error (sd). Student's unpaired $t$-test $^{**}P < 0.005$.

G Western blot analysis of wild type (ctrl) and TFEB;3$^{KO}$ clones, showing the absence of TFEB and TFE3 proteins. β-tubulin was used as a loading control. Asterisk indicates non-specific band.

H FACS analysis of DQ-BSA dye fluorescence in RCS with indicated genotypes (ctrl = wild type) treated with FGF18 (50 ng/ml for 16 h). Fluorescence intensities were calculated as % relative to vehicle (5% ABS). Mean ± standard error of the mean (SEM) of $N = 4$ biological replicates. One-way analysis of variance (ANOVA) $P = 0.024$.; Tukey's post hoc test $^{*}P < 0.05$; NS, not significant.

I qRT–PCR analysis of lysosomal genes in wild type (ctrl) and TFEB;3$^{KO}$ RCS treated with FGF18 (50 ng/ml for 16 h). Fold change values were relative to vehicle and normalized to *Cyclophilin* gene. Mean ± standard error of the mean (SEM) of $N = 4$ biological replicates. Analysis of variance (ANOVA) CTSA $P = 0.0003$; CTSD $P = 0.0065$; Lamp1 $P = 0.00002$; Tukey's post hoc test $^{**}P < 0.005$; $^{*}P < 0.05$.

J qRT–PCR analysis of lysosome genes expression in chondrocytes with indicated genotypes (ctrl = wild type). TFEB-S142A:S211A and TFE3-S246A:S312A mutant plasmids were overexpressed for 48 h; FGF18 (50 ng/ml) treatment was for 16 h. Values were normalized to *Cyclophilin* gene and expressed as fold change relative to cells transfected with empty vector (mock). Mean ± standard error of the mean (SEM) of $N = 3$ biological replicates. One-way analysis of variance (ANOVA) Tukey's post hoc test $^{**}P < 0.005$.

K Immunofluorescence staining of Lamp1 (red) in RCS overexpressing TFE3-S246A:S312A-GFP and TFEB-S142A:S211A-GFP (green) with indicated genotype. Insets show lysosomes. TFEB and TFE3 expressing cells have smaller and less vacuolized lysosomes. Nuclei were stained with DAPI (blue). Scale bar 10 and 2 μm (higher magnification boxes). Representative images of $N = 3$ biological replicates.

Source data are available online for this figure.

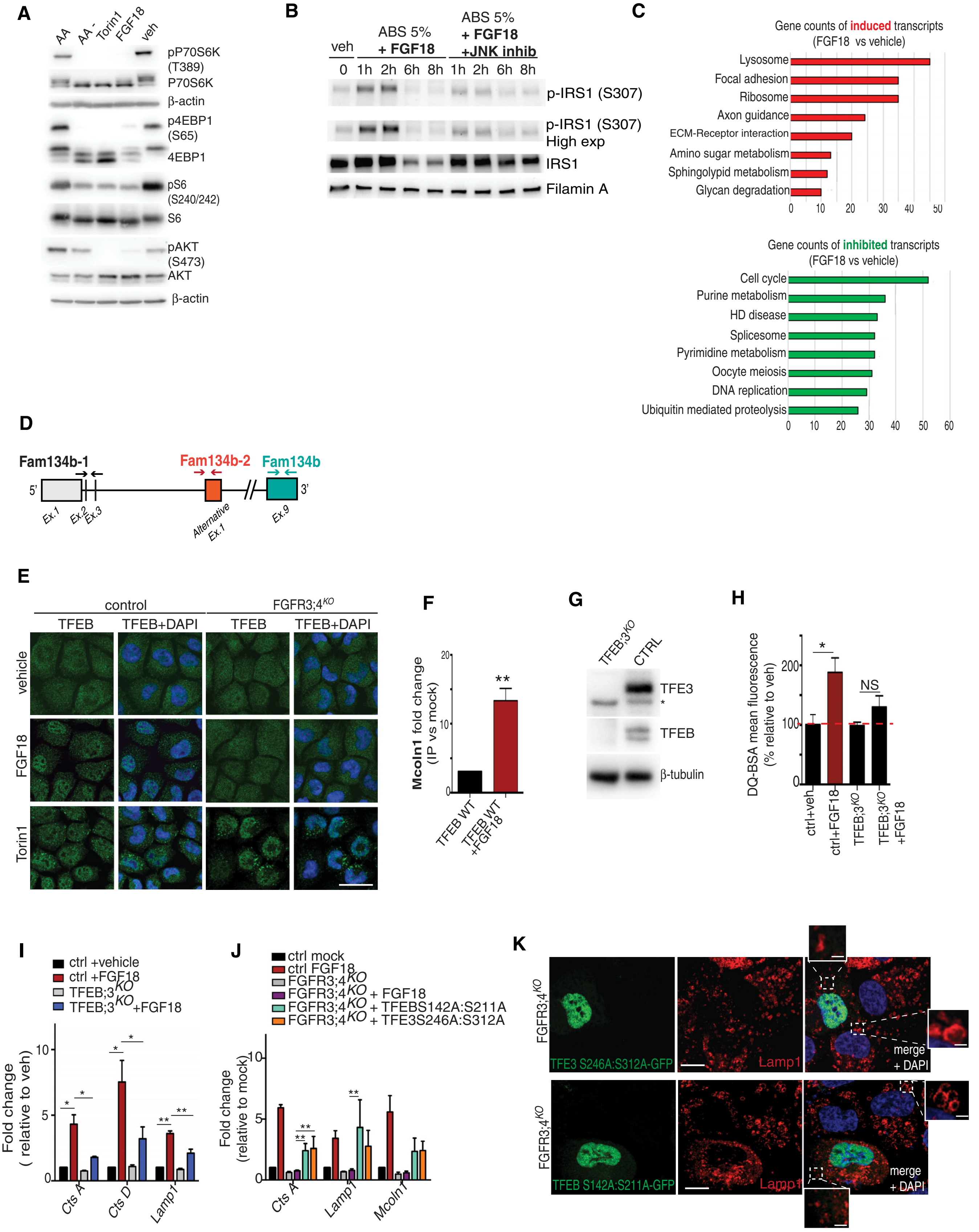


**Figure EV2.**

 © 2020 The Authors

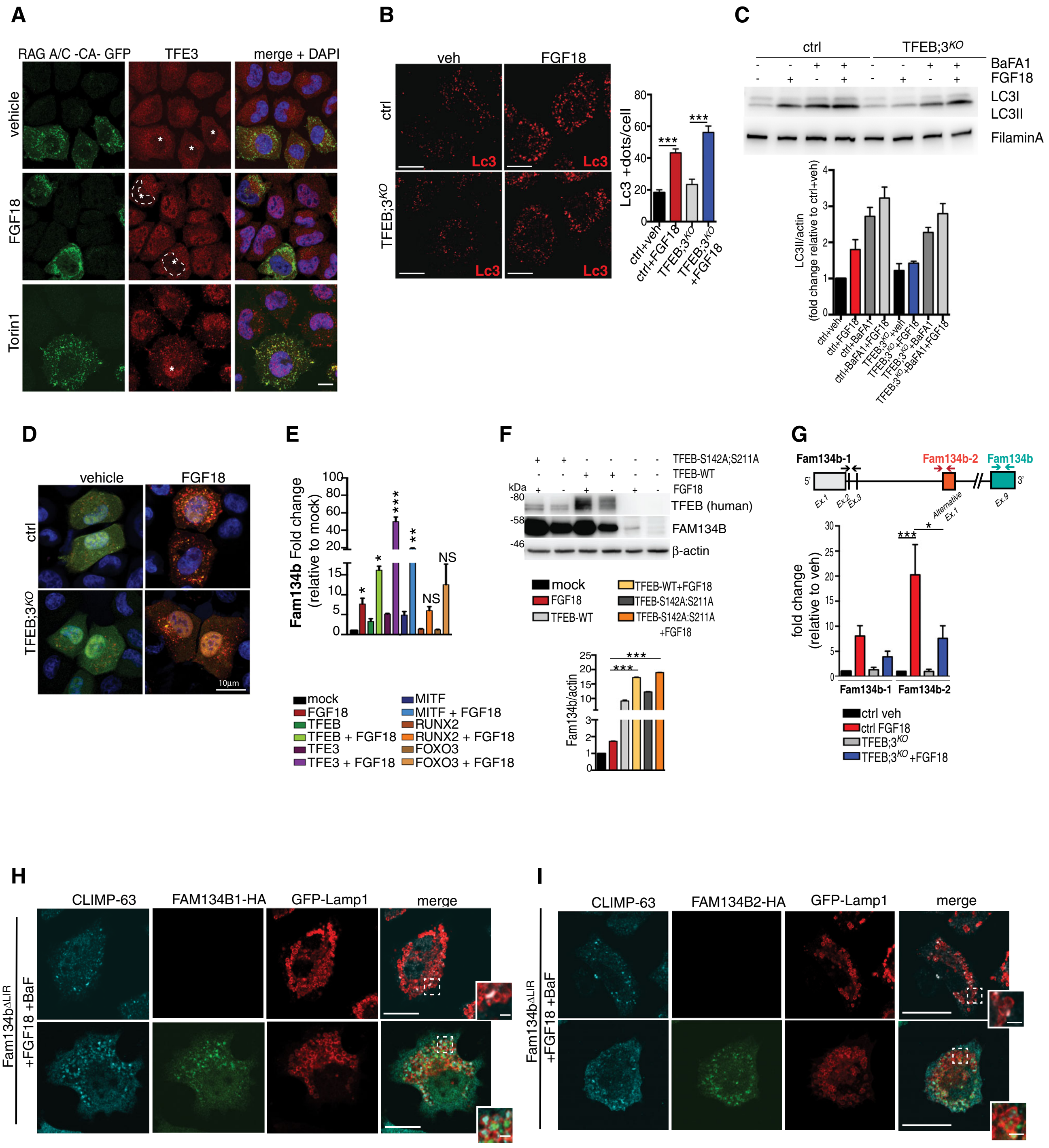
A
RAG A/C -CA- GFP
TFE3
merge + DAPI
vehicle
FGF18
Torin1
B
veh
FGF18
ctrl
TFEB;3KO
Lc3
Lc3 +dots/cell
ctrl+veh
ctrl+FGF18
TFEB;3KO
TFEB;3KO +FGF18
C
ctrl
TFEB;3KO
BaFA1
FGF18
LC3I
LC3II
FilaminA
LC3II/actin (fold change relative to ctrl+veh)
D
vehicle
FGF18
ctrl
TFEB;3KO
10µm
E
Fam134b Fold change (relative to mock)
NS
mock
FGF18
TFEB
TFEB + FGF18
TFE3
TFE3 + FGF18
MITF
MITF + FGF18
RUNX2
RUNX2 + FGF18
FOXO3
FOXO3 + FGF18
F
TFEB-S142A;S211A
TFEB-WT
FGF18
kDa
TFEB (human)
FAM134B
β-actin
mock
FGF18
TFEB-WT
TFEB-WT+FGF18
TFEB-S142A:S211A
TFEB-S142A:S211A +FGF18
Fam134b/actin
G
Fam134b-1
Fam134b-2
Fam134b
Alternative Ex.1
fold change (relative to veh)
ctrl veh
ctrl FGF18
TFEB;3KO
TFEB;3KO +FGF18
H
CLIMP-63
FAM134B1-HA
GFP-Lamp1
merge
Fam134bΔLIR +FGF18 +BaF
I
CLIMP-63
FAM134B2-HA
GFP-Lamp1
merge
Fam134bΔLIR +FGF18 +BaF

**Figure EV3.**

**Figure EV3. FGF18 induces Fam134b expression and ER-phagy.**

A Representative immunofluorescence staining of TFE3 (red) in RAGA/C-CA-GFP (green) overexpressing RCS treated with FGF18 (50 ng/ml overnight) and Torin 1 (1 μM for 2 h). RAGA/C-CA-GFP overexpressing cells (asterisks) retain TFE3 in the cytoplasm in FGF18 (empty nuclei were highlighted by dashed white lines), but not in Torin 1 treatment. Nuclei were stained with DAPI (blue). Scale bar 10 μm.

B Representative images of immunofluorescence staining of LC3 protein in wild type (ctrl) and TFEB;3$^{KO}$ RCS treated with vehicle (5% ABS) and FGF18 (50 ng/ml) for 16 h. Scale bar 10 μm. Quantification of LC3-positive dots/cell. FGF18 was used at 50 ng/ml for 16 h. $N$ = 17 cells/genotypes were analyzed. Student's paired $t$-test ***$P$ < 0.005.

C Western blot analysis of LC3B in wild type (ctrl) and TFEB;3$^{KO}$ RCS chondrocytes treated with vehicle (5% ABS) and FGF18 (50 ng/ml) for 12 h. BafA1 (200 nM; 4 h) was used to inhibit lysosome activity. β-actin was used as a loading control. Blots are representative of $N$ = 4 independent experiments. Bar graph shows quantification of LC3II normalized to β-actin. Mean ± standard error of the mean (SEM).

D Representative immunofluorescence staining of mCherry-GFP-LC3 tandem experiment assay in wild type (ctrl) and TFEB;3$^{KO}$ RCS treated with vehicle (5% ABS) and FGF18 (50 ng/ml) for 12 h. Nuclei were stained with DAPI (blue).

E qRT–PCR analysis of *Fam134b* gene expression in RCS transfected with indicated transcription factors for 48 h. FGF18 (50 ng/ml; 16 h) was added where indicated. Values were normalized to *Cyclophilin* gene and expressed as fold change relative to mock-transfected cells. Mean ± standard error of the mean (SEM) of $N$ = 4 biological replicates/treatment. Student's paired $t$-test *$P$ < 0.05 and one-way analysis of variance (ANOVA) $P$ = 0.0001; Tukey's post hoc test ***$P$ < 0.0005; **$P$ < 0.005; NS, not significant.

F Western blot analysis of FAM134B and TFEB proteins in RCS overexpressing TFEB-WT and TFEB-S142A:S211 plasmids for 48 h. FGF18 was used at 50 ng/ml for 16 h. β-actin was used as a loading control. Representative images of $N$ = 3 biological replicates. Bar graph shows quantification of Fam134b normalized to β-actin. Mean ± standard error of the mean (SEM). Student's paired $t$-test ***$P$ < 0.0005.

G Schematic representation of Fam134b DNA locus. Arrows indicate the positions qPCR primer pairs used to detect Fam134b-1 (black arrows), Fam134b-2 (brown arrows), or both (turquoise arrows) isoforms. qRT–PCR of *Fam134b-1* and *Fam134b-2* isoforms in RCS with indicated genotypes (veh and FGF18 refer to treated wild-type chondrocytes). FGF18 (50 ng/ml; 16 h) was added where indicated. Values were normalized to *Cyclophilin* gene and expressed as relative to mock-transfected cells. Mean ± standard error of the mean (SEM) of $N$ = 4 (*Fam134b-1*) and $N$ = 5 (*Fam134b-2*) biological replicates/treatment. One-way analysis of variance (ANOVA) $P$ = 0.0013; Sidak's multiple comparison test ***$P$ < 0.0005; *$P$ < 0.05.

H, I Immunofluorescence staining of CLIMP63 (light blue) and lysosomes (GFP-Lamp1, red) in FAM134bΔLIR RCS chondrocytes transfected with Fam134b-1 (H) and Fam134b-2 (I) HA-tagged (green) plasmids upon FGF18 treatment (50 ng/ml for 16 h). BafA1 (100 nM; 4 h) was used to inhibit lysosomal degradation. Insets show magnification of CLIMP63 accumulation into lysosomes. Scale bar 10 and 2 μm (higher magnification boxes).

Source data are available online for this figure.

**Figure EV4. Starvation induces ER-phagy in RCS and HeLa cells through TFEB and TFE3.**

A Western blot analysis of phospho-P70S6K (T389), P70S6K, and phospho-TFEB (S142) in RCS cultured in complete medium or HBSS for 16 h. β-actin was used as a loading control. Bar graph shows quantification of phospho-TFEB (S142) normalized to β-actin. Mean ± standard error of the mean (SEM) of $N$ = 3 biological replicates. Student's paired $t$-test *$P$ < 0.05.

B Subcellular localization analysis of TFE3 (green) in RCS chondrocytes starved with HBSS for 8 h. Torin 1 was used at 1 μM for 2 h as positive control. Nuclei were stained with DAPI (blue). Quantification analysis showed % of cells with nuclear TFEB and TFE3 in RCS with indicated treatments. Mean ± standard error of the mean (SEM) of $N$ = 3 biological replicates/treatment. Scale bar 10 μm. $n$ = 62 cells (control), 83 cells (HBSS), 47 cells (Torin 1). One-way analysis of variance (ANOVA) $P$ < 0.0001; Sidak's multiple comparison test ***$P$ < 0.0005.

C qRT–PCR analysis of *Fam134b* isoforms in wild type (ctrl) and TFEB;3$^{KO}$ RCS. Cells were starved overnight with HBSS where indicated. Values were normalized to *Cyclophilin* gene and expressed as fold change relative to control (complete medium). Mean ± standard error of the mean (SEM) of $N$ = 3 biological replicates. One-way analysis of variance (ANOVA) $P$ < 0.0001; Sidak's multiple comparison test ***$P$ < 0.0005; NS, not significant.

D EATR assay in chondrocytes with indicated genotypes (ctrl = wild type) showing % of cells with acidified ER measured by FACS analysis. HBSS starvation was for 16 h. Mean ± standard error of the mean (SEM) of $N$ = 3 biological replicates/treatment/genotype. One-way analysis of variance (ANOVA) $P$ < 0.0001; Sidak's multiple comparison test ***$P$ < 0.0005; *$P$ < 0.05.

E Co-staining of CLIMP63 (green) and TMEM192-HA (lysosomes, red) in chondrocytes with indicated genotypes (ctrl = wild type) and starved with HBSS for 16 h where indicated. BaFA1 was used at 100 nM for 4 h. Scale bar 10 and 2 μm (higher magnification boxes). Bar graph shows quantification of relative CLIMP63 fluorescence in TMEM192-HA decorated lysosomes. Mean ± standard error of the mean (SEM) of $N$ = 3 biological replicates. $N$ = 10 cells/experiment were analyzed. One-way analysis of variance (ANOVA) $P$ < 0.0001; Sidak's multiple comparison test ***$P$ < 0.0005.

F Co-staining of CLIMP63 (green) and TMEM192-HA (lysosomes, red) in wild type (ctrl) and TFEB;3$^{KO}$ HeLa cells with indicated genotypes (ctrl = wild type) and starved with HBSS for 16 h where indicated. BaFA1 was used at 100 nM for 4 h. Scale bar 15 and 2 μm (higher magnification boxes). Quantification showed CLIMP63 relative fluorescence in TMEM192-HA decorated lysosomes. Mean ± standard error of the mean (SEM) of $N$ = 3 biological replicates. $N$ = 26 (HeLa full media), $N$ = 29 (HeLa HBSS), $N$ = 22 (HeLa TFEB;3$^{KO}$ full media), $N$ = 25 (HeLa TFEB;3$^{KO}$ HBSS) cells were analyzed. Student's paired $t$-test *$P$ < 0.05; NS, not significant.

G EATR assay in HeLa cells with indicated genotypes (ctrl = wild type) showing % of cells with acidified ER measured by FACS analysis. HBSS starvation was for 16 h. Mean ± standard error of the mean (SEM) of $N$ = 5 biological replicates/treatment/genotype. One-way analysis of variance (ANOVA) $P$ = 0.0002; Sidak's multiple comparison test **$P$ < 0.005; NS, not significant.

Source data are available online for this figure.

 © 2020 The Authors

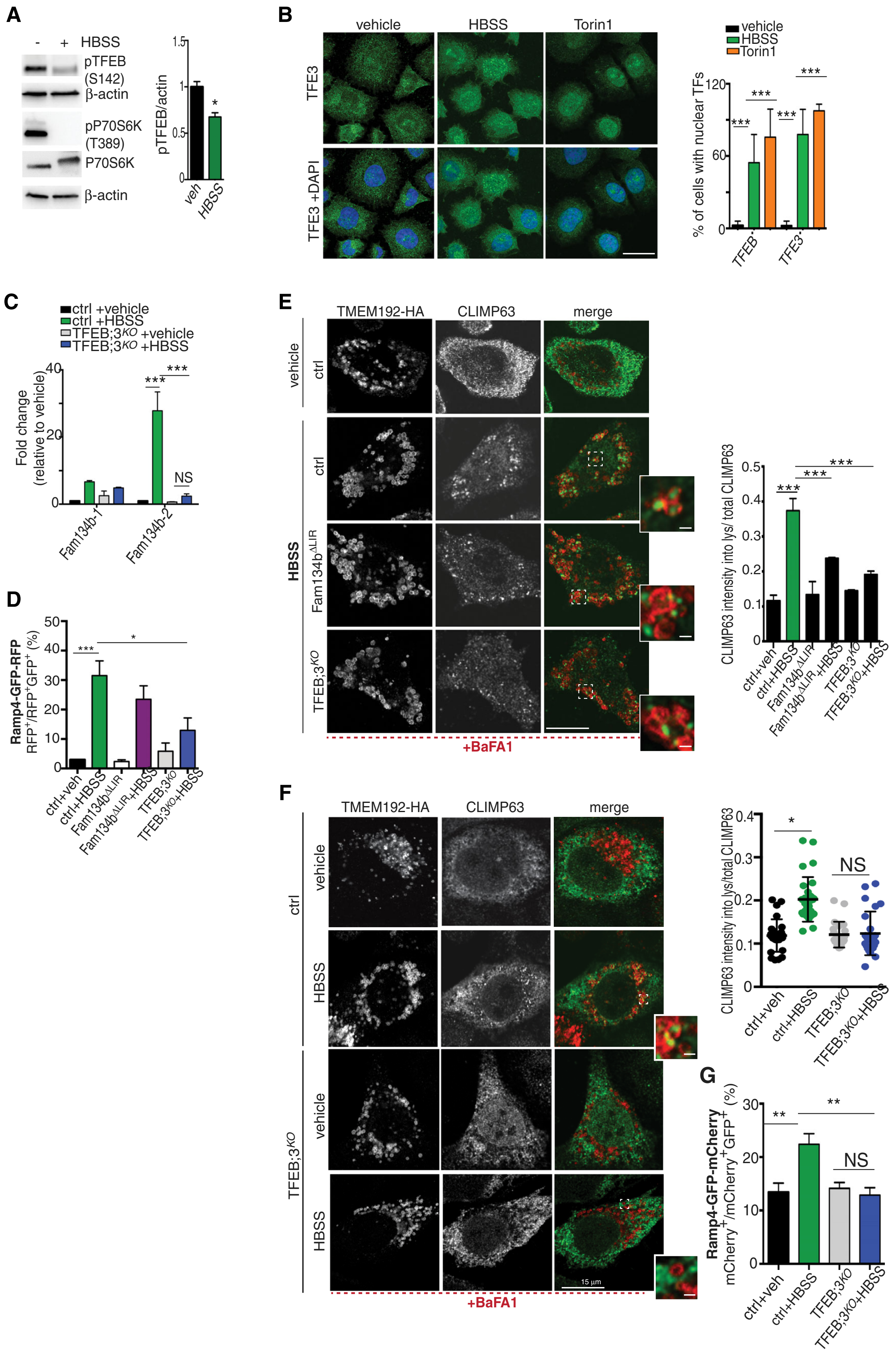
A
- + HBSS
pTFEB (S142)
β-actin
pP70S6K (T389)
P70S6K
β-actin
pTFEB/actin
veh
HBSS
B
vehicle
HBSS
Torin1
TFE3
TFE3 +DAPI
% of cells with nuclear TFs
TFEB
TFE3
C
ctrl +vehicle
ctrl +HBSS
TFEB;3KO +vehicle
TFEB;3KO +HBSS
Fold change (relative to vehicle)
NS
Fam134b-1
Fam134b-2
D
Ramp4-GFP-RFP RFP+/RFP+GFP+ (%)
ctrl+veh
ctrl+HBSS
Fam134bΔLIR
Fam134bΔLIR+HBSS
TFEB;3KO
TFEB;3KO+HBSS
E
TMEM192-HA
CLIMP63
merge
vehicle
ctrl
HBSS
Fam134bΔLIR
TFEB;3KO
+BaFA1
CLIMP63 intensity into lys/ total CLIMP63
F
ctrl
TFEB;3KO
NS
15 µm
G
Ramp4-GFP-mCherry mCherry+/mCherry+GFP+ (%)

**Figure EV4.**

**Figure EV5. Fam134b-HA mRNA expression rescued F*am134b*$^{mo}$ skeletal phenotype.**

A qRT–PCR analysis of *Fam134b* gene in medaka fish with indicated genotypes. Values were normalized to *Hprt* gene and expressed as fold change relative to scramble fish. Mean ± standard error of the mean (SEM) of *N* = 3 biological replicates.

B Reverse transcription–PCR analysis of *Fam134b* gene in *Fam134b*$^{wt}$ and *Fam134b*$^{mo}$ medaka fish. Actin gene was used as control gene. M = marker; B = blank.

C Western blot analysis of HA-tag from a pool of medaka fish embryos injected with scramble or injected with mRNA produced from human HA-FAM134B pcdna3.1 (+). β-actin was used as a loading control.

D Bar graphs show quantification of total length and head size of medaka fish model of F*am134b*$^{mo}$ and mRNA-injected F*am134b*$^{mo}$ expressed as % relative to the scramble. Mean ± standard error of the mean (SEM) of at least *n* = 8 fish per genotype. Student's unpaired *t*-test *$P < 0.05$; NS, not significant.

E, F Bar graphs show quantification of Alcian Blue (cartilage) (e) and Alizarin Red (bone) (f) staining of *Fam134b*$^{mo}$ and mRNA-injected F*am134b*$^{mo}$. Ethmoid plate (EP), palatoquadrate (PQ), ceratohyal (CH), paired prootics (PO), ceretobranchials 1–5 (CB1 to CB5) cartilage length (E), and bone mineralization (F) were evaluated. Values were expressed as % relative to the scramble (100% red dotted line). Mean ± standard error of the mean (SEM) of at least *n* = 6 fish/genotype. Student's unpaired *t*-test *$P < 0.05$; **$P < 0.005$; ***$P < 0.0005$ for comparison between F*am134b*$^{mo}$ and mRNA-injected F*am134b*$^{mo}$ medaka.

Source data are available online for this figure.


© 2020 The Authors

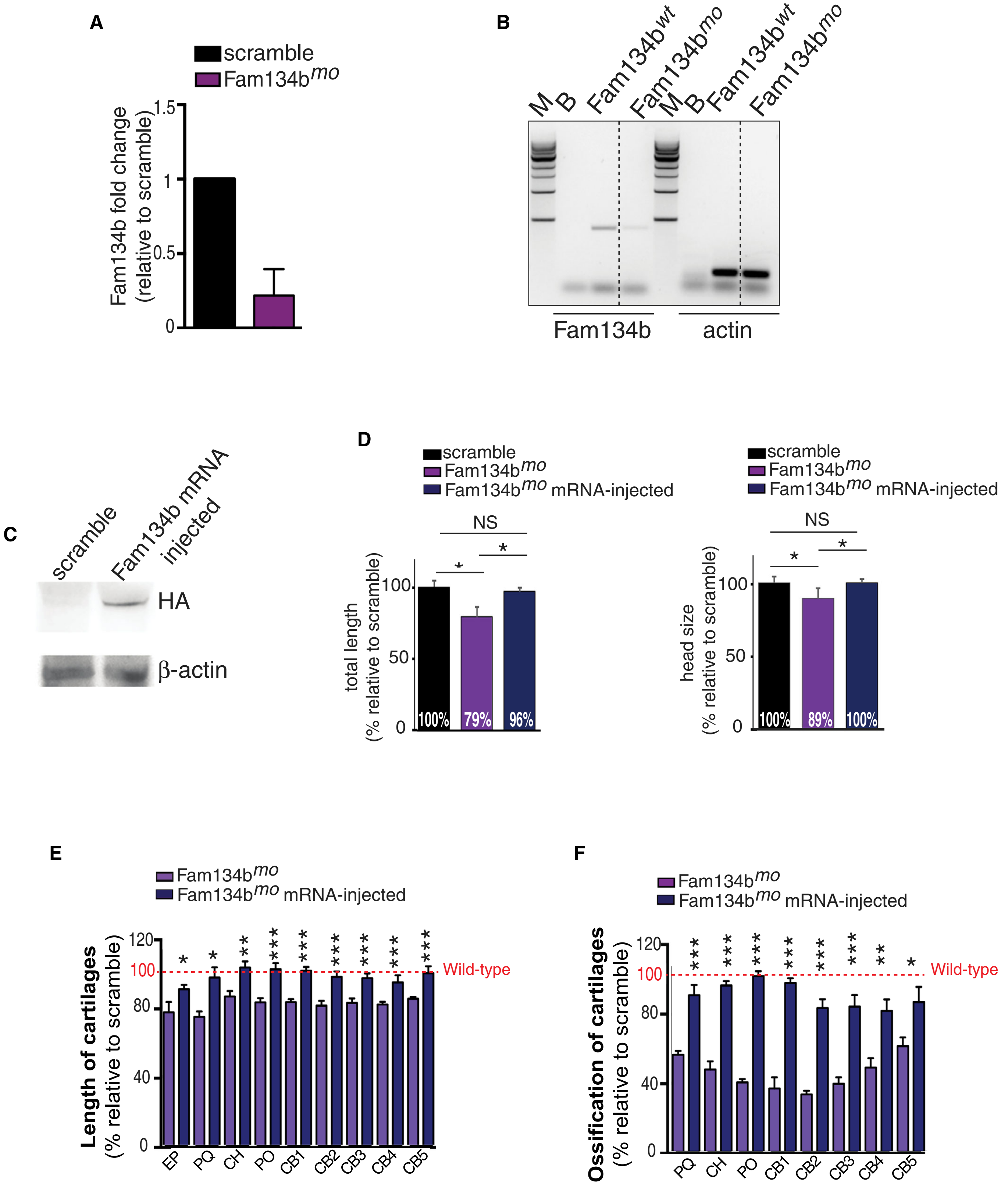
A
scramble
Fam134b mo
Fam134b fold change (relative to scramble)
B
M B Fam134b wt Fam134b mo M B Fam134b wt Fam134b mo
Fam134b
actin
C
scramble
Fam134b mRNA injected
HA
β-actin
D
scramble
Fam134b mo
Fam134b mo mRNA-injected
NS
total length (% relative to scramble)
100%
79%
96%
head size (% relative to scramble)
89%
E
Length of cartilages (% relative to scramble)
Wild-type
EP PQ CH PO CB1 CB2 CB3 CB4 CB5
F
Ossification of cartilages (% relative to scramble)

**Figure EV5.**
