# Supplementary figures and images for: MiT/TFE factors control ER‐phagy via transcriptional regulation of FAM134B
Source: EMBO J. 2020 Jul 27;39(17):e105696. doi: 10.15252/embj.2020105696 (PMC7459426; doi:10.15252/embj.2020105696)

EV Figure 3C

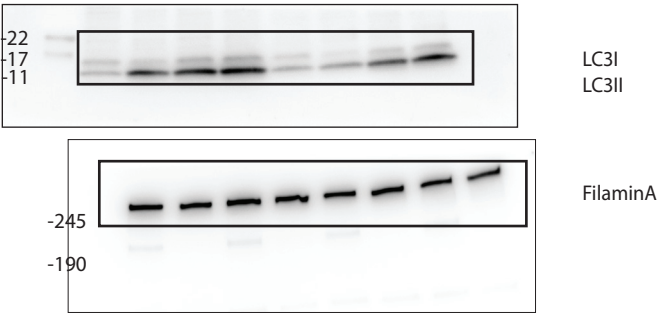

EV Figure 3F

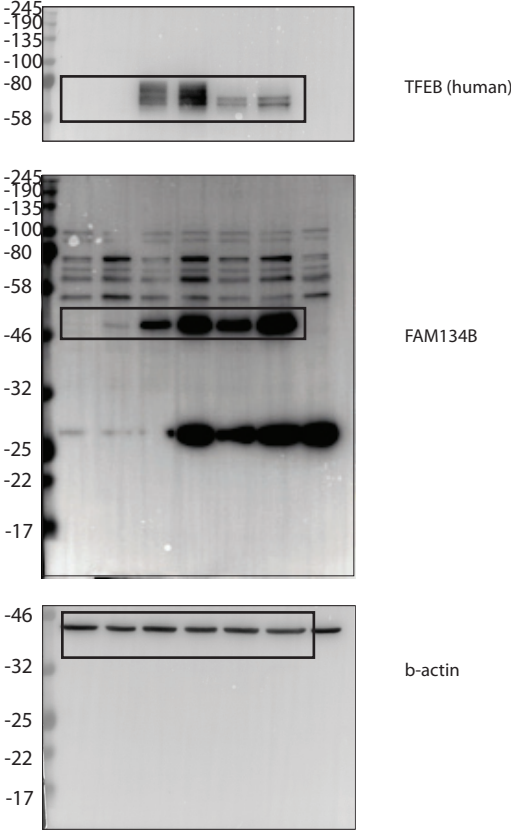

Supplement: Supplementary file 10 — Source Data for Expanded View [file EMBJ-39-e105696-s017.zip › source data EV figure 3_19-6-20.pdf]

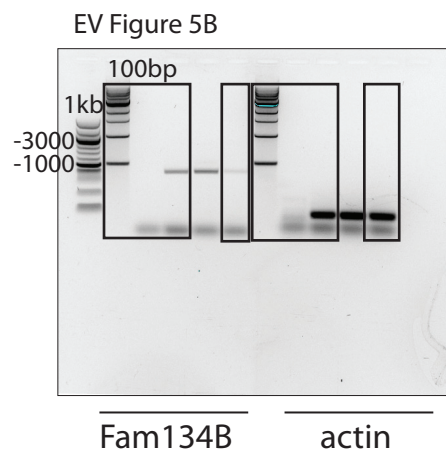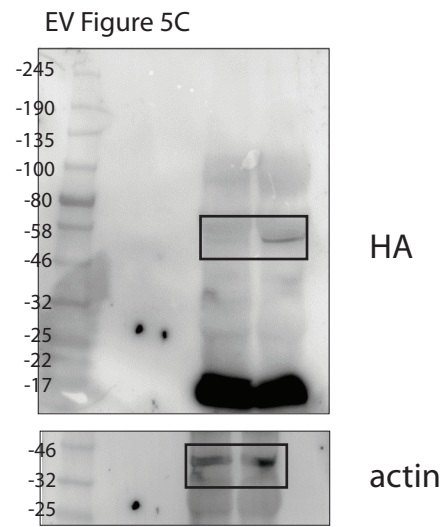

Supplement: Supplementary file 10 — Source Data for Expanded View [file EMBJ-39-e105696-s017.zip › source data EV figure 5_19-6-20.pdf]

EV Figure 2A

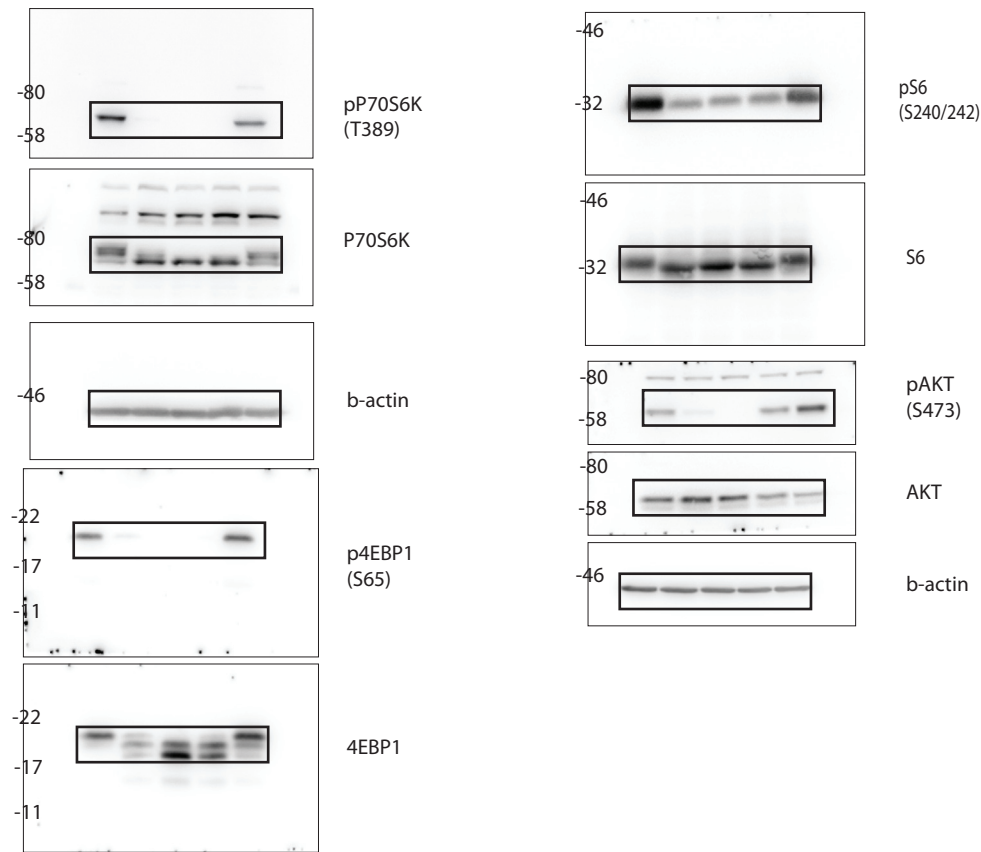

EV Figure 2B

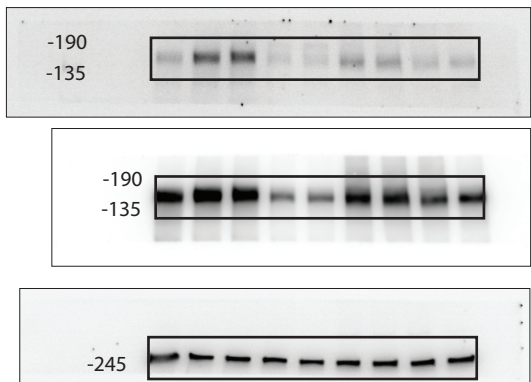

EV Figure 2G

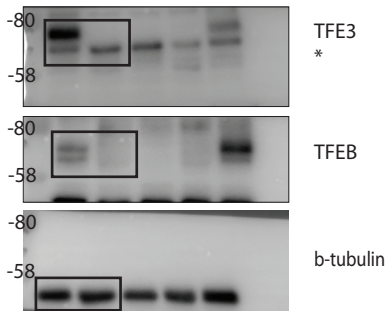

Supplement: Supplementary file 10 — Source Data for Expanded View [file EMBJ-39-e105696-s017.zip › source data EV figure 2_19-6-20.pdf]

EV Figure 4A

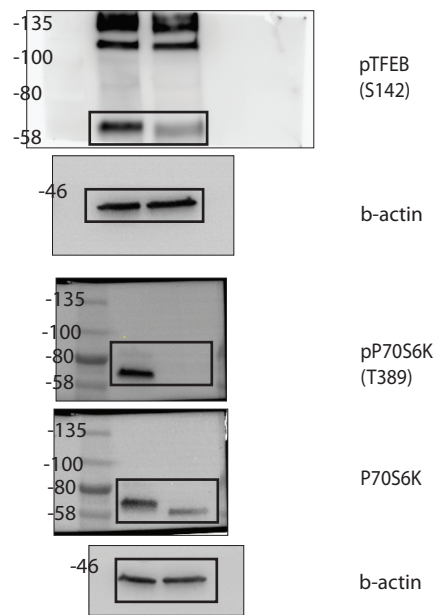

Supplement: Supplementary file 10 — Source Data for Expanded View [file EMBJ-39-e105696-s017.zip › source data EV figure 4_19-6-20.pdf]

SOURCE DATA EV FIGURE 1

EV Figure 1G

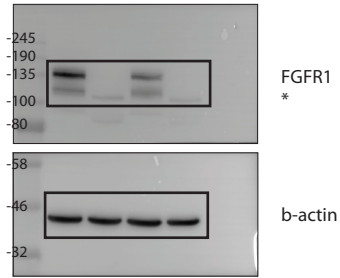

EV Figure 1G

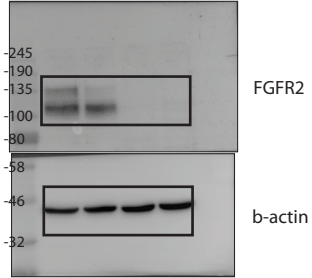

EV Figure 1G

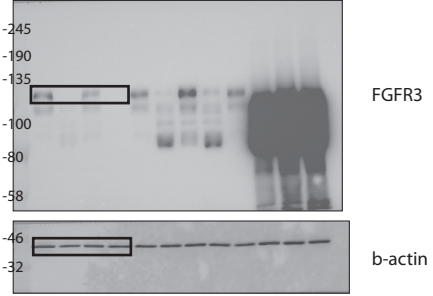

EV Figure 1G

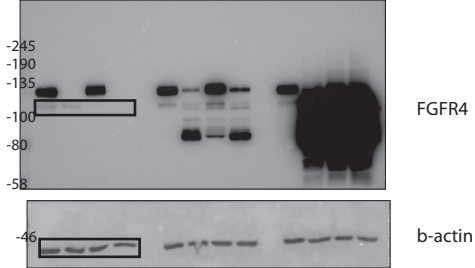

EV Figure 1G

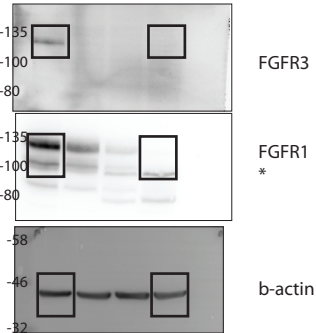

EV Figure 1E

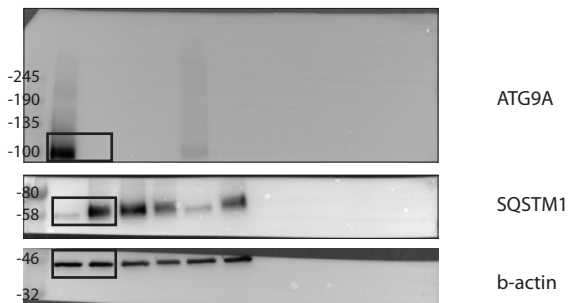

Supplement: Supplementary file 10 — Source Data for Expanded View [file EMBJ-39-e105696-s017.zip › source data EV figure 1_19-6-20.pdf]

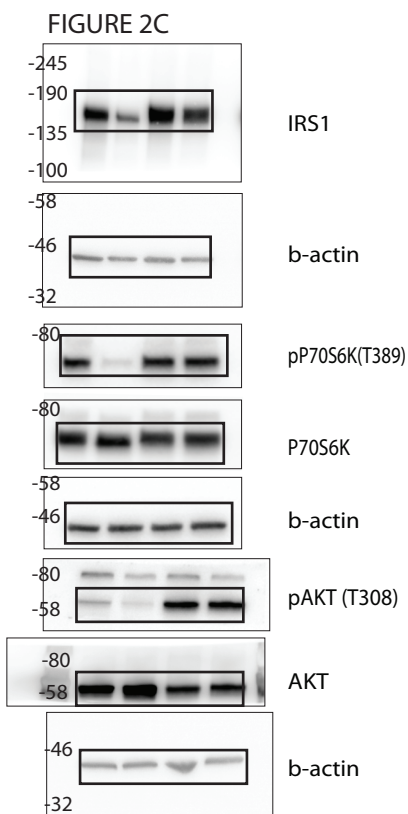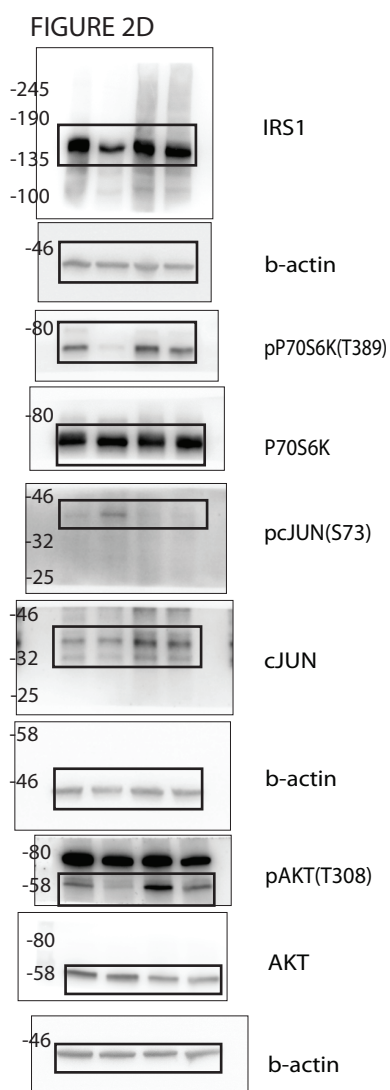

Supplement: Supplementary file 12 — Source Data for Figure 2 [file EMBJ-39-e105696-s010.pdf]

FIGURE 3F

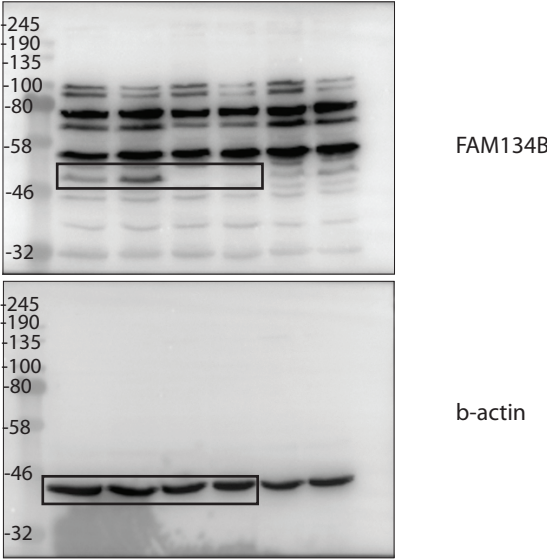

Supplement: Supplementary file 13 — Source Data for Figure 3 [file EMBJ-39-e105696-s011.pdf]

FIGURE 4B

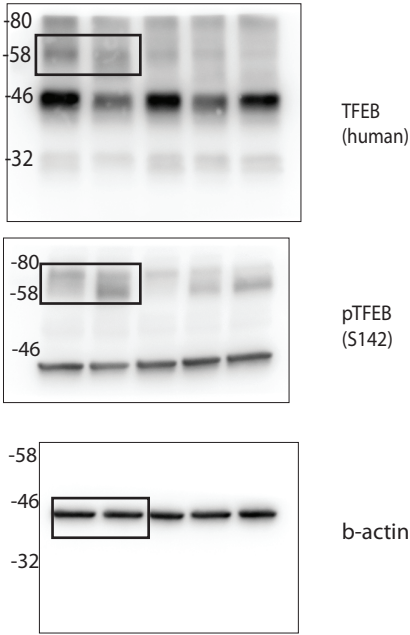

FIGURE 4F

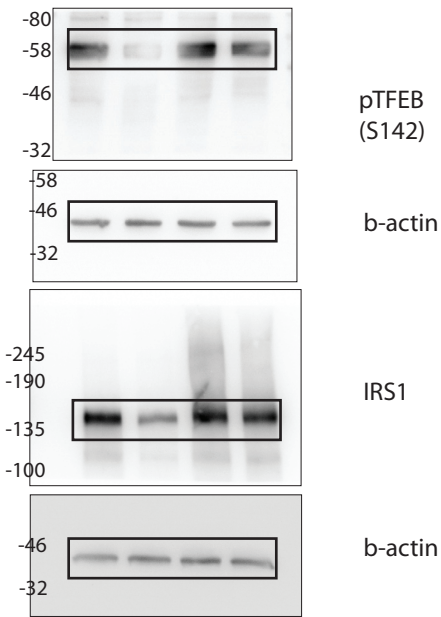

Supplement: Supplementary file 14 — Source Data for Figure 4 [file EMBJ-39-e105696-s012.pdf]

FIGURE 5B

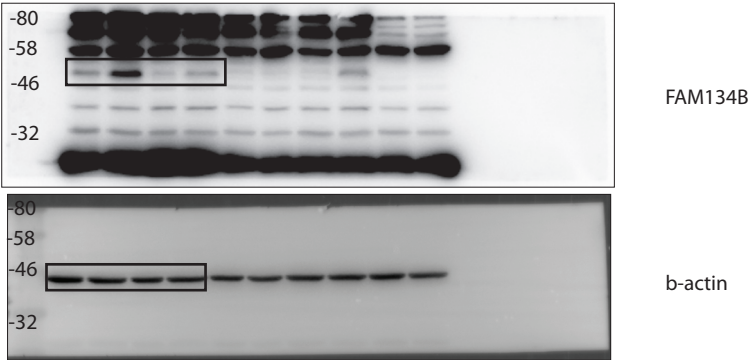

Supplement: Supplementary file 15 — Source Data for Figure 5 [file EMBJ-39-e105696-s013.pdf]

Figure 6B

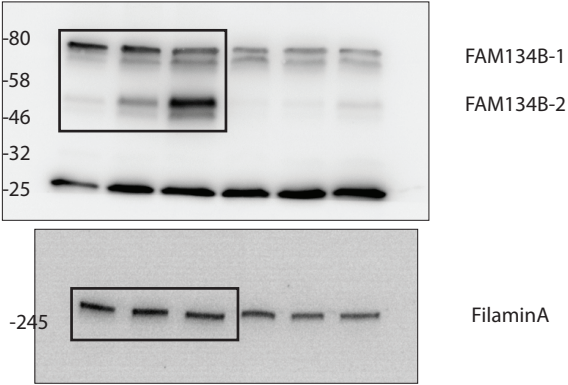

Supplement: Supplementary file 16 — Source Data for Figure 6 [file EMBJ-39-e105696-s014.pdf]

FIGURE 7D

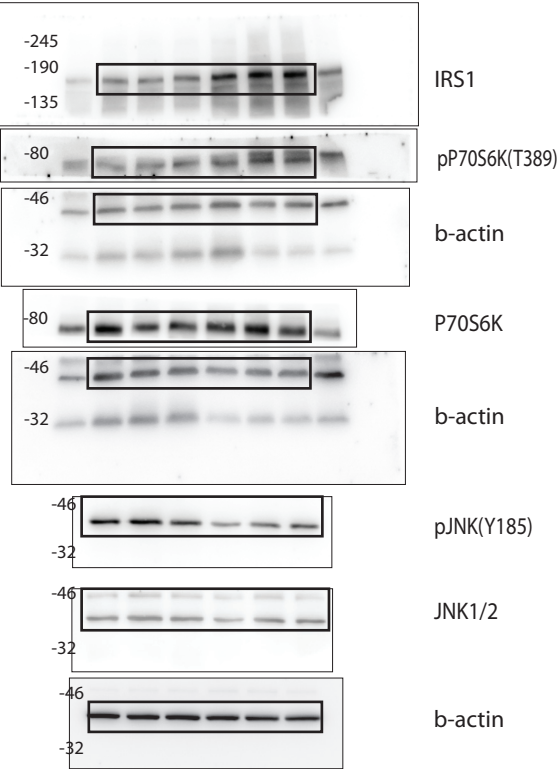

FIGURE 7E

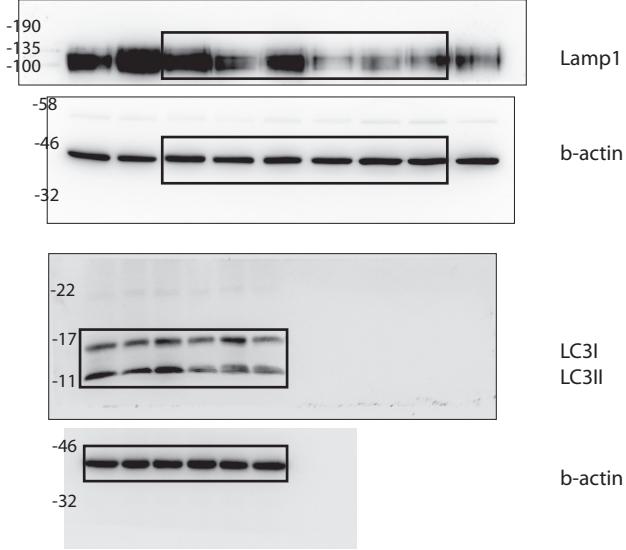

FIGURE 7G

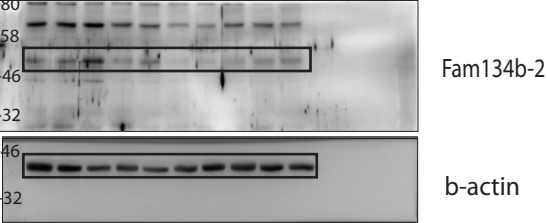

Supplement: Supplementary file 17 — Source Data for Figure 7 [file EMBJ-39-e105696-s015.pdf]

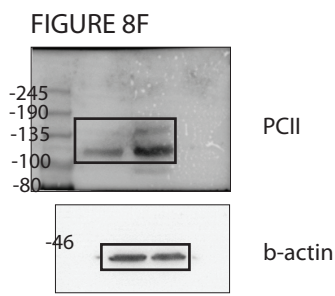

Supplement: Supplementary file 18 — Source Data for Figure 8 [file EMBJ-39-e105696-s016.pdf]
